# 17**α**-Ethynyl-androst-5-ene-3**β**,7**β**,17**β**-triol (HE3286) Is Neuroprotective and Reduces Motor Impairment and Neuroinflammation in a Murine MPTP Model of Parkinson's Disease

**DOI:** 10.1155/2012/969418

**Published:** 2012-09-26

**Authors:** Ferdinando Nicoletti, Ingrid Philippens, Paolo Fagone, Clarence N. Ahlem, Christopher L. Reading, James M. Frincke, Dominick L. Auci

**Affiliations:** ^1^Department of Biomedical Sciences, School of Medicine, University of Catania, Via Androne 81, 95124 Catania, Italy; ^2^Department of Immunobiology, Biomedical Primate Research Centre, Lange Kleiweg 161, 2288 GJ Rijswijk, The Netherlands; ^3^Harbor Therapeutics, Inc., 9191 Towne Centre Drive, Suite 409, San Diego, CA 92122, USA; ^4^Tumor Vaccine Group, School of Medicine, University of Washington, Seattle, WA 98109, USA

## Abstract

17**α**-Ethynyl-androst-5-ene-3**β**,7**β**,17**β**-triol (HE3286) is a synthetic androstenetriol in Phase II clinical development for the treatment of inflammatory diseases. HE3286 was evaluated for blood-brain barrier (BBB) permeability in mice, and efficacy in a 1-methyl-4-phenyl-1,2,3,6-tetrahydropyridine (MPTP) murine model of Parkinson's disease (PD). We found that HE3286 freely penetrated the BBB. HE3286 treatment significantly improved motor function compared to vehicle in the rotarod test (mean 58.2 sec versus 90.9 sec, *P* < 0.0001), and reduced inflammatory mediator gene expression in the brain (inducible nitric oxide synthase, 20%, *P* = 0.002; tumor necrosis factor **α**, 40%, *P* = 0.038, and interleukin-1**β**, 33%, *P* = 0.02) measured by reverse-transcriptase polymerase chain reaction. Brain tissue histopathology and immunohistochemistry showed that HE3286 treatment increased the numbers of tyrosine hydroxylase-positive cells by 17% compared to vehicle (*P* = 0.003), and decreased the numbers of damaged neurons by 38% relative to vehicle (*P* = 0.029). L-3,4-dihydroxyphenylalanine (**L**-DOPA) efficacy was not enhanced by concurrent administration of HE3286. HE3286 administration prior to MPTP did not enhance efficacy. Our data suggest a potential role for HE3286 in PD treatment, and provides incentive for further investigation.

## 1. Introduction

PD is a neurodegenerative disorder characterized by a progressive degeneration of dopaminergic (DAergic) neurons in the substantia nigra pars compacta (SNpc) and decreased levels of dopamine in the putamen of the dorsolateral striatum. The loss of dopamine in the striatum manifests clinically as motor disabilities that include bradykinesia, resting tremor, and muscular rigidity. Diagnosis is based on motor symptoms, which become evident only after the loss of more than 50% of the SNpc DAergic neurons and 60–80% of striatal dopamine [1]. Prolonged treatment of PD with **L**-DOPA usually results in a dyskinesia that can be more disabling than the disease itself; therefore, there is a great need for alternative therapeutic modalities. The acute MPTP mouse model of nigrostriatal degeneration recapitulates the DAergic neuron loss seen in PD and currently represents the most commonly used toxin-induced mouse model of PD [2]. MPTP's mechanism of toxicity is complex, and exerted through its toxic metabolite, methyl-4-phenylpyridinium (MPP+) ion, which is taken up selectively by DAergic neurons through the dopamine transporter. Inside the cell, MPP+ is a mitochondrial toxin, which induces neuronal death through several mechanisms that include oxidative stress [3], excitotoxicity [4], and inflammation [5]. Recent publications have shown that neuroinflammation mediated by extracellular signal regulated kinase (ERK1/2) can contribute to excitotoxicity through hyperactivation of NMDA receptors, increasing Ca^+2^ flux and thereby, increasing nitric oxide (NO) radical damage and mitochondrial stress through mechanisms complementary to the direct actions of MPP+. Interestingly, NMDA receptor hyperactivation can also activate ERK [6, 7] suggesting the potential for NMDA receptor activation to feedforward. The inflammatory cascade is further promoted by microglia activation by NO and other inflammatory mediators from astrocytes and distressed neurons. Neuroinflammation has been known to be associated with PD for decades, but only recently has the causal relationship between inflammation and neurodegeneration been appreciated in PD and other neurodegenerative diseases. By virtue of sterile inflammatory mediators, cells outside the immune system can initiate, propagate, or respond to inflammatory stimuli [8, 9]. These stimuli can act alone, or in concert with the immune system, to produce inflammation in the absence of an infectious pathogen [10]. Uncovering the mechanisms of sterile inflammation has been central to clarifying the pathophysiology of progressive neurodegeneration [11, 12]. The elucidation of inflammatory mechanisms in PD suggests the potential use of anti-inflammatory agents for disease modification.

HE3286 is an orally-active synthetic derivative of the anti-inflammatory dehydroepiandrosterone (DHEA) metabolite, androstene-3*β*,7*β*,17*β*-triol (*β*AET). HE3286 is pharmacologically unrelated to glucocorticoids and sex steroids, and although anti-inflammatory, it is not immunosuppressive and has no apparent potential for systemic toxicity at pharmacologically relevant exposures in rodents and canines [13]. *β*AET is found naturally in picomolar concentrations in human plasma [14], but is virtually absent in rodents without exogenous DHEA administration. *β*AET is not orally bioavailable, and plasma concentrations in humans cannot be appreciably augmented by DHEA supplementation due to DHEA's unfavorable absorption and metabolism characteristics [15]. HE3286 has anti-inflammatory activity in rodent disease models [16, 17]. Both *β*AET and HE3286 display many of the desirable anti-inflammatory activities reported for DHEA [18], suggesting that some aspects of DHEA's activity stem from polyhydroxylated metabolites that are readily formed in rodents [15]. HE3286 has no known intrinsic neuropharmacological activity or toxicity [13]. HE3286 binds ERK1/2, low-density lipoprotein receptor-related protein 1 (Lrp1) and sirtuin 2 (Sirt2) [19], and HE3286 decreases ERK1/2 activation in lipopolysaccharide-(LPS)-stimulated rodent macrophages [20]. HE3286 is a Phase II clinical development compound with a good safety profile in humans to date (manuscript submitted). Because HE3286 binds and regulates ERK1/2, and ERK1/2 appears to play a critical role in neurodegenerative diseases [21], a demonstration of HE3286 activity in a model of PD would provide rationale for clinical studies in PD and other neurodegenerative diseases.

In the present studies, we first tested the ability of HE3286 to cross the blood-brain barrier (BBB) and then evaluated HE3286's motor skill and neuron preservation activity in an acute MPTP mouse model of PD. We found that HE3286 readily permeated the BBB and that HE3286 treatment of MPTP-induced animals significantly improved motor function, reduced neuroinflammation, and decreased neurodegeneration relative to vehicle treated controls. Our data suggest the potential for HE3286 to treat PD.

## 2. Methods

### 2.1. Reagents

HE3286 was obtained by custom synthesis from Norac Pharma (Azusa, CA, USA), and was formulated for oral gavage as an aqueous microsuspension in 1 mg/mL carboxymethyl cellulose sodium salt, 9 mg/mL NaCl, 20 mg/mL polysorbate-80, and 0.5 mg/mL phenol. All excipients were obtained from Spectrum Chemicals (New Brunswick, NJ, USA). MPTP and **L**-DOPA were purchased from Sigma Aldrich (Milan, Italy). 

### 2.2. Animal Care and Use

All animal studies were conducted in accordance with the *Guide for the Care and Use of Laboratory Animals* as adopted and promulgated by the US National Institutes of Health.

### 2.3. Evaluation of BBB Permeability

Twenty-one, 6–8-week-old male CD-1 mice (Charles River Laboratories, Wilmington, MA, USA) received a single oral gavage of 80 mg/kg HE3286 aqueous microsuspension (4 mL/kg). Cohorts of three mice were sacrificed after 0.5, 1, 2, 3, 4, 8, and 24 hours by cardiac puncture under CO_2_ anesthesia. Blood was processed to serum and stored frozen until analyzed. After blood collection, the brains from each mouse were collected, snap frozen, and stored frozen until analyzed. Each brain was weighed and homogenized using an ultrasonic cell disrupter; the homogenates were centrifuged to remove debris, and processed as serum for HE3286 liquid chromatography-mass spectrometry/mass spectrometry (LC-MS/MS) analysis.

### 2.4. HE3286 Quantitation by LC-MS/MS

Serum and brain samples were spiked with internal standard (17*α*-methyl-androst-5-ene-3*β*,7*β*,17*β*-triol) and extracted with methyl tert-butyl ether. The organic portions were evaporated, reconstituted in liquid chromatography mobile phase, and analyzed on a Waters Xbridge Phenyl column by reversed-phase, high-performance liquid chromatography (Agilent, Palo Alto, CA, USA and Leap Technologies, Carrboro, NC, USA) coupled with a tandem quadrupole mass spectrometer (Waters, Beverly, MA, USA). Calibration curves for standards and quality control samples for HE3286 were prepared with the samples. Sample responses were acquired and concentrations were determined based on the calibration using MassLynx analysis software (Waters, Beverly, MA, USA). The lower limit of quantification was 1 ng/mL. Samples with concentrations below the quantifiable limit were assigned a value of zero.

### 2.5. Disease Induction and Drug Administration

HE3286 activity was evaluated in three independent experiments. Eight-to-ten-week-old C57/Bl6 male mice (Harlan Laboratories S.r.l., Udine, Italy) were housed under standard laboratory conditions with free access to food and water for one week prior to randomization into groups of eight. In the first experiment, neurotoxicity was initiated in three groups of mice (groups 2, 3, and 4) with four intraperitoneal injections of 20 mg/kg MPTP two hours apart, while one group (group 1) received saline injections to provide a normal control, and group 2 received no treatment after MPTP. One hour after the last MPTP or saline injection, a four-day treatment schedule was initiated: group 3 received vehicle gavage twice daily (BID), and group 4 received HE3286 40 mg/kg gavage BID.

In two subsequent experiments, two additional groups of MPTP-treated mice (groups 5 and 6) were added to the protocol. Both groups received a twice-daily, 4-day therapeutic regimen beginning the day before MPTP induction; group 5 received **L**-DOPA (24.8 mg/kg per dose injected intraperitoneally [i.p.], based on a mean body weight of 20 g) plus vehicle by gavage, and group 6 received **L**-DOPA i.p., and 40 mg/kg HE3286 per dose by gavage. Groups 1–4 of these experiments were treated as described for experiment 1.

### 2.6. Motor Coordination Measurements

Before MPTP injection, mice were trained at several different rod rotation speeds, and motor skill performance was calculated as the mean latency to fall from the rotating rod (Ugo Basile, Comerio, Italy). Baseline motor coordination in the rotarod test was recorded for each mouse 24 h after training was completed (24 h before MPTP injection.). Four days after MPTP injection, hypokinesia-like symptoms were assessed by a constant speed (20 RPM) rotarod test. 

After-MPTP testing consisted of three trials (180 s) with an intertrial interval of 30 min. Test performance was defined as the mean of the three trials. Mice were tested starting 1.5 h after the last therapeutic treatment.

After completing the rotarod test, the animals were euthanized by CO_2_ asphyxiation. The brains were collected and segments were allocated for gene expression and histological analysis.

### 2.7. Semiquantitative Polymerase Chain Reaction

Brain sections for gene expression measurements were stored in RNAlater solution (Applied Biosystems, Foster City, CA, USA) at 4°C until total RNA was extracted using TRIzol reagent (Invitrogen, Grand Island, NY, USA), according to manufacturer's instructions. RNA quality was evaluated by measuring the 260/280 nm absorbance ratio (≥1.8) and by electrophoresis. Complementary DNA (cDNA) was synthesized using 1 *μ*g of total RNA using the TaqMan retrotranscription reagents as described by the manufacturer (Applied Biosystems, Foster City, CA, USA). Polymerase chain-reaction (PCR) was carried out in a 30 *μ*L final volume containing 200 nM forward and 200 nM reverse primers and 20 ng of cDNA. The following primer pairs were used, inducible nitric oxide synthase (iNOS) FWD: AATCTTGGAGGGAGTTGTGG; iNOS REV: CAGGAAGTAGGTGAGGGTTTG; tumor necrosis factor alpha (TNF-*α*) FWD: AGCCCACGTCGTAGCAAACCACCA; TNA-*α* REV: ACACCCATTCCCTTCACAGAGCAA; interleukin-1 beta (IL-1*β*) FWD: ACACTCCTTCGTCCTCGGCCA; IL-1*β* REV: CCATCAGAGGCAAGGAGGAA; glyceraldehyde phosphate dehydrogenase (GAPDH) FWD: CTAGAGAGCTGACAGTGGGTAT; (GAPDH) REV: AGACGACCAATGCGTCCAAA. The amplified fragments were run in a 1% agarose gel and densitometric analysis was performed using Image J software. Gene expression data are presented as the ratio between target gene expression and GAPDH control gene expression. 

### 2.8. Histology

Brain segments for histopathology were fixed in 4% paraformaldehyde for 1 week, embedded in paraffin, and sliced in 5 *μ*m-thick sections, which were stained with hematoxylin–eosin (H&E) to visualize cell bodies. The number of damaged neurons was obtained as an average of cells counted per field. The general criteria to score damaged cells included hyperchromatic nuclei and cytoplasmic vacuolation. The number of damaged neurons was visually estimated on three sections from four animals for each experimental group.

### 2.9. Immunohistochemistry

At the end of the intervention, mice to be analyzed by immunohistochemistry were anesthetized and transcardially perfused with paraformaldehyde (4% in 0.1 M phosphate buffer, pH 7.4). Coronal sections of the SNpc were cut on a vibratome, stained with antityrosine hydroxylase (TH) antibodies (polyclonal rabbit anti-TH, 1 : 1000, Abcam), biotinylated secondary antibodies (goat anti-rabbit IgG for TH, Abcam, Cambridge, UK), and avidin-horseradish peroxidase conjugate according to the manufacturer's instructions (ABC, Vector, Peterborough, UK). The image was developed with 3,3′-diaminobenzidine (Sigma, Milan, Italy) as the chromogen. TH positive neurons were enumerated on three serial sections per animal. TH-labelled neurons were scored as positive only if their cell body image included well-defined nuclear counterstaining. The number of TH positive neurons was determined in blinded fashion by an independent pathologist.

### 2.10. Statistical Evaluation

Data from all experiments were combined for analysis and tested for normal distribution using the Shapiro-Wilks *W* test. Normally distributed data were analyzed by Student's *t*-test. Data that were not normally distributed were analysed by the nonparametric Mann-Whitney test. For multiple comparisons, the Kruskal-Wallis test was used to determine overall significance, with Dunn's post hoc test for significance of groups compared to control. Statistical significance for all tests was ascribed to *P* ≤ 0.05. All statistical tests were carried out using GraphPad Prism 5 (GraphPad Software Inc., San Diego, CA).

## 3. Results

### 3.1. HE3286 Blood-Brain Barrier Penetration

**Figure 1:**
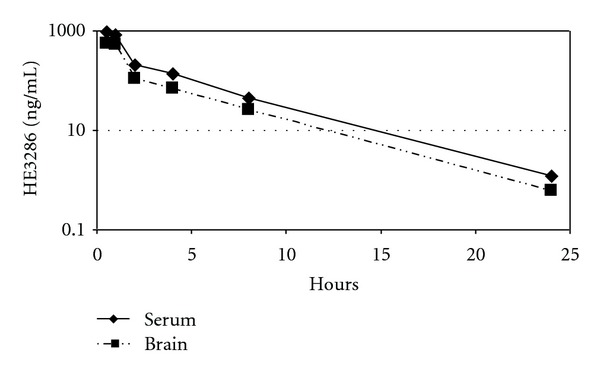
HE3286 readily penetrates the BBB. CD-1 mice (males, *n* = 3 per group) were treated (gavage) with HE3286 (80 mg/kg HE3286) and sacrificed at 0.5, 1, 2, 3, 4, 8, and 24 h. Serum and brain samples were analyzed for HE3286 by LC-MS/MS. Each point represents the mean value derived from three animals.

The kinetics of HE3286 in serum and brain were parallel indicating that HE3286 moved freely across the BBB (Figure 1). The mean ratio of the drug concentration in brain relative to serum from 0.5 to 24 h was  0.571 ± 0.084, which was attained even in the earliest sample collected.

## 4. Evaluation of Efficacy in the MPTP Model

### 4.1. Mortality

HE3286 was tested three times in the MPTP model, and in each experiment mortalities were observed the day after MPTP induction. In the first experiment (8 per group), 2, 1, and 2 mice were found dead in the MPTP, MPTP + vehicle, and MPTP + HE3286 groups, respectively. In the second and third experiments combined (16 total per group), 3, 1, 1, 1, and 3 mice were found dead in the MPTP, MPTP + vehicle, MPTP + HE3286, MPTP + **L**-DOPA + vehicle, and MPTP + **L**-DOPA + HE3286 groups, respectively. No deaths were observed in saline treated animals (group 1).

### 4.2. Effect of HE3286 Treatment on MPTP-Induced Motor Impairment

**Figure 2:**
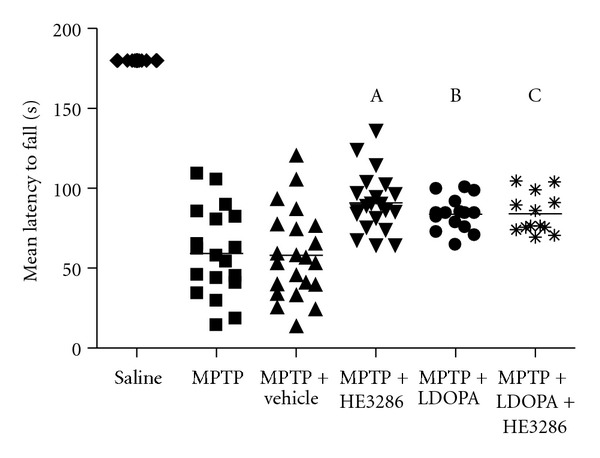
HE3286 effect on motor impairment in experiments 1–3 combined. Mice (male, C57/Bl6 mice, 8 per group) were given four injections of 20 mg/kg MPTP 2 h apart. **L**-DOPA (24.8 mg/kg i.p.) and **L**-DOPA (i.p.) in combination with HE3286 (40 mg/kg gavage) were administered for 4 d beginning 1 h prior to MPTP, or HE3286 (40 mg/kg), vehicle or saline was administered by gavage twice daily for four consecutive days beginning 1 h after the last MPTP injection. Motor impairment was measured using the rotorod test (A, *P* < 0.0001 compared to vehicle, B, *P* = 0.0016, C, *P* = 0.0031).

We evaluated the effect of HE3286 on MPTP-induced motor coordination impairment by measuring the ability of animals to balance on a rotating cylinder (rotarod test). The combined results for the rotarod test for experiments 1, 2, and 3 are summarized in Table 1 and presented graphically in Figure 2. The baseline mean latency to fall for all the mice before MPTP injection was 180.0 ± 0.0 sec (all mice remained on the rod until the end of the test). MPTP treatment reduced this value to 59.2 ± 27.3 and 58.2 ± 27.5 sec in the absence of any treatment or vehicle treatment, respectively.

Treatment with HE3286 after MPTP induction significantly increased the mean latency to fall to 90.9 ± 18.4 sec (*P* < 0.0001 versus vehicle).

The mean latency to fall was 83.8 ± 10.6 sec (*P* = 0.0016 versus vehicle) for treatment with **L**-DOPA (initiated prior to MPTP). The mean latency for the combination of **L**-DOPA with HE3286 initiated prior to MPTP was 84.0 ± 12.5 sec (*P* = 0.0031 versus vehicle), similar to HE3286 initiated after MPTP induction. There was no significant difference between HE3286 + **L**-DOPA compared to their respective effects as monotherapies (*P* > 0.1 Mann-Whitney). 

### 4.3. Effect of HE3286 Treatment on MPTP-Induced Neuroinflammation

**Figure 3:**
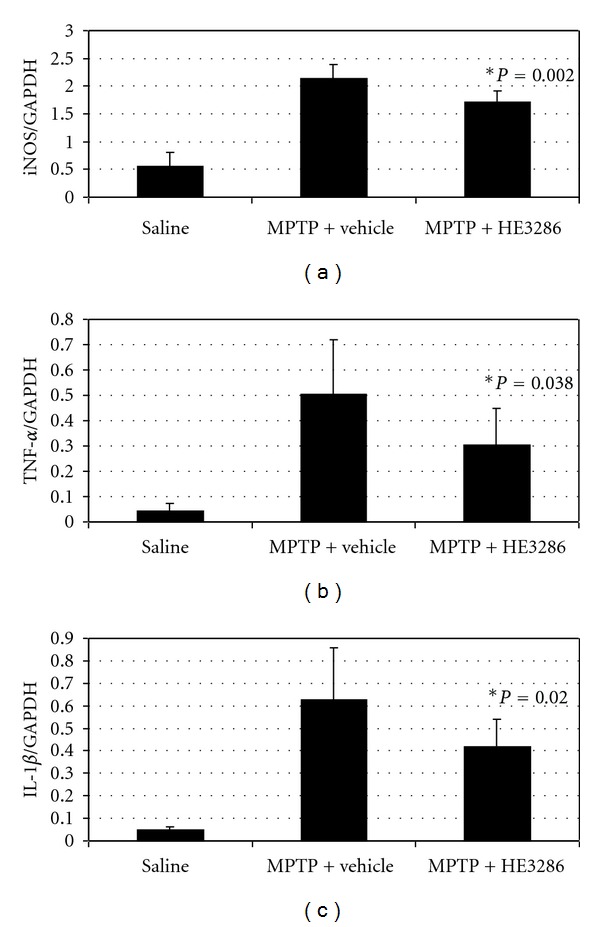
HE3286 attenuated MPTP-induced expression of inflammatory cytokines. Mice (male, C57/Bl6 mice, 8 per group) received four injections of 20 mg/kg MPTP 2 h apart. HE3286 (40 mg/kg), vehicle, or saline was administered by oral gavage twice-daily for four consecutive days beginning 1 h after the last MPTP injection. Gene expression for iNOS (a), TNF-*α* (b), and IL-1*β* (c) was assessed by semiquantitative reverse transcriptase-polymerase chain reaction. Gene expression data are presented as the ratio between target gene expression and GAPDH control gene expression. *Statistically significant compared to MPTP + vehicle by the Mann-Whitney test.

**Figure 4:**
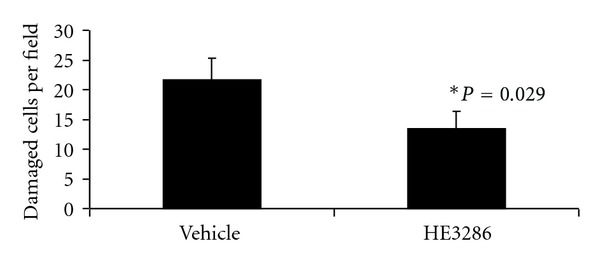
HE3286 decreased MPTP-induced neuronal damage. Mice (male, C57/Bl6 mice, 8 per group) were given four injections of 20 mg/kg MPTP 2 h apart. HE3286 (40 mg/kg), vehicle or saline was administered by oral gavage twice daily for four consecutive days beginning 1 h after the last MPTP injection. Sections from fixed brain segments from four randomly selected animals per group were stained with H&E and the numbers of damaged neurons per field were counted. *Significant compared to MPTP + vehicle by the Mann-Whitney exact test.

In order to elucidate the therapeutic effects of HE3286, inflammation and neuronal damage were assessed in control and treated animals using PCR and histology. As expected, naïve control mice expressed low levels of the proinflammatory mediators, iNOS, TNF-*α*, and IL-1*β* (Figure 3), while MPTP-induced vehicle-treated mice expressed high mRNA levels. HE3286 significantly reduced iNOS, TNF-*α*, and IL-1*β* RNA expression (*P* < 0.05). Histology revealed a significant (*P *= 0.01) reduction in the number of damaged neurons in the mice treated with HE3286 compared to vehicle treated animals (Figure 4).

### 4.4. Effect of HE3286 on MPTP-Induced TH-Positive Neuron Loss in the SNpc

**Figure 5:**
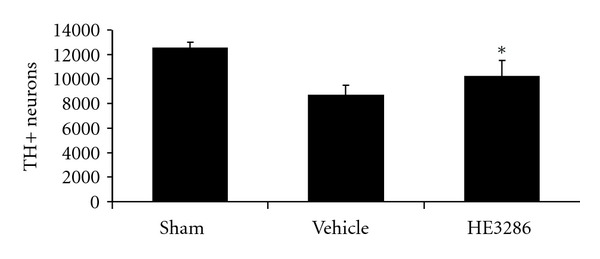
HE3286 attenuated TH-positive neuron loss in the SNpc. Mice (male, C57/Bl6, 8 per group) were given four injections of 20 mg/kg MPTP 2 h apart. HE3286 (40 mg/kg), vehicle, or saline was administered by oral gave twice daily for four consecutive days beginning 1 h after the last MPTP injection. Four days after MPTP injection, mice were sacrificed and brains collected. Coronal SNpc sections from four randomly selected animals per group were immunoreacted with anti-TH antibodies. TH positive neurons were determined on three serial sections per animal. *Significantly greater compared to vehicle, *P* = 0.003; significantly lower compared to sham, *P* = 0.0024, by the Mann-Whitney exact test.

MPTP treatment reduced the number of dopaminergic neurons in the SNpc (Figure 5). Treatment with HE3286 significantly attenuated TH-positive neuron loss in the SNpc (*P* < 0.003 versus vehicle). 

## 5. Discussion

The present experiments were performed to determine if HE3286's anti-inflammatory activity would translate to neuroprotective activity in a rodent model of PD. We found that HE3286 readily permeated the BBB, and that oral administration of HE3286 decreased the loss of motor coordination induced by MPTP, and decreased the expression of inflammatory mediators in the brains of MPTP-treated mice. Importantly, HE3286 attenuated the loss of TH-positive neurons and decreased neuron damage. The absence of an enhanced therapeutic effect from HE3286 treatment prior to MPTP (group 6) suggests that HE3286 has no direct activity against MPTP mitochondrial toxicity.

Although some inflammatory conditions can compromise the BBB, providing hydrophilic or high molecular weight drug access to the central nervous system (CNS), BBB permeability and the potential for P-glycoprotein-driven efflux are otherwise important considerations in CNS drug screening [22, 23]. HE3286 was rapidly exchanged between systemic circulation and the CNS. The saline: octanol partition coefficient (LogD) of HE3286 is 1.3 (Harbor Therapeutics, unpublished); others have shown that molecules within the LogD range of 0.9–2.3 are freely permeable to baboon brain [24]. The high concentration of drug achieved in the brain relative to the concentration in the blood suggests that HE3286 is not a substrate for P-glycoprotein mediated efflux, noting the same conclusion was derived from investigations of HE3286 interactions with P-glycoprotein *in vitro* (Harbor Therapeutics, unpublished).

HE3286 had a significant effect on motor coordination skills, which was similar in magnitude to **L**-DOPA treatment. Unfortunately, **L**-DOPA concentrations in the SNpc were not measured in our experiments. This data might have provided functional confirmation of the cells preserved by HE3286, which we determined solely by the expression TH. We presume that our observation of HE3286's equivalence to **L**-DOPA supplementation (in terms of effect on motor coordination) resulted from augmentation of endogenous **L**-DOPA by preservation of TH+ cells, but we cannot exclude other mechanisms, such as decreased hyperactivation of NMDA receptors through anti-inflammatory effects. One may speculate that enhanced endogenous **L**-DOPA would not cause **L**-DOPA-induced dyskinesia (LID), but it may be difficult to address this question without considering the possible influence of HE3286 on LID through anti-inflammatory mechanisms [25]. The potential to delay LID onset would have enormous benefit, and should be investigated.

In the MPTP model used for the present experiments, neurodegeneration occurs gradually over the course of several days [26, 27] as a consequence of inflammatory excitotoxicity subsequent to the initial mitochondrial insult [28], and is therefore suitable to evaluate a candidate anti-inflammatory therapy for PD [5, 12]. However, chronic neuroinflammation, which is central to PD, cannot be fairly studied in this acute model, especially within the 4-day treatment schedule in our experiments. 

An eight-hour course of MPTP treatment increased iNOS, TNF*α*, and IL-1*β* expression 3-, 20-, and 13-fold, respectively. All three of these proteins are important factors in neurodegeneration, so the observed reductions in expression by HE3286 are relevant to HE3286's neuroprotective activity (assuming protein concentrations are reflected by RNA abundance, since RNA results were not corroborated with protein measurements.) Nitric oxide radical products of iNOS are important mediators of MPTP mitochondrial toxicity [29], while IL-1*β* [30] and TNF*α* [31] are linked to NMDA receptor hyperactivation and excitotoxicity. The present experiments were not designed to measure the kinetics of the cytokine flux, but HE3286 anti-inflammatory activity is observable within 15 minutes *in vitro* [20], and in 24 hours in other *in vivo* models [32].

HE3286 ERK binding [19] and regulation of ERK activation [20] may be responsible for the anti-inflammatory and neuroprotective effects observed in the current study. MPTP initiates a neuroinflammatory sequence involving (among other inflammatory mediators) high mobility group box 1 (HMGB1) that prolongs ERK activation [11]. In neurons, ERK has a complex and context-dependent function that regulates long-term memory [33] and pro or anticell survival mechanisms [34, 35], and ERK is an early central inflammatory signal mediator in a neurodegenerative signalling cascade [36]. Efforts to pharmaceutically intervene with ERK activation must recognize ERK's involvement in critical cell functions as well as pathologies. Other investigators have suggested that a partial reduction in ERK activation, which allowed normal function, may be a useful approach to reduce the negative consequences of ERK hyperactivation, while supporting homeostasis [25]. ERK hyperactivation through toll-like receptors (TLR) and the receptor for advanced glycation end products (RAGE) has been linked to neuronal apoptosis and progressive neurodegeneration in PD [36]. ERK upregulates NMDA receptor expression and NR2B subunit phosphorylation at tyrosine residues 1252, 1336, or 1472 through a series of signalling events involving CREB and src activation [37]. NR2B phosphorylation increases neuronal Ca^+2^ flux and potentiates neuronal hyperexcitability and excitotoxicity [37]. An evaluation of ERK activation with HE3286 treatment in the MPTP model will help to clarify the relationship between drug target and our proposed mechanism of action. We suggest that HE3286 as an ERK regulator, but not a classical inhibitor, may possess a useful therapeutic index for the treatment of neuroinflammatory disease. Indeed, extensive toxicology studies with HE3286 have provided no evidence of a neurological side effect [13]. It is interesting to note that ERK hyperactivation also has a pivotal roll in other neuroinflammatory conditions such as epilepsy [37], chronic pain [38], Alzheimer's disease [39], and importantly in the context of this study, **L**-DOPA, induced dyskinesia [25].

The preservation of both motor skills and DAergic neurons in the MPTP model observed in the present studies, in combination with a favourable pharmaceutical profile [13] suggest a potential role for HE3286 in the treatment of PD and parkinsonian-related disorders.

## Figures and Tables

**Table 1:** ANOVA showed a significant effect overall (Kruskal-Wallis, *P* = 0.0003). Dunn's post hoc test indicated significance for MPTP + HE3286 and MPTP +L-DOPA compared to MPTP + vehicle, *P* < 0.0001 and *P* < 0.05, respectively. There was no improvement with MPTP + HE3286 + L-DOPA compared to MPTP + L-DOPA (*P* > 0.05 by the Mann-Whitney test).

Group	*n*		Mean rotarod latency (sec)	SD	95% CI	*P* versus MPTP + vehicle^a^
	Expts	Mice				
Saline	3	24	180	0	180-180	
MPTP	3	19	59.2	27.3	46–72	
MPTP + vehicle	3	22	58.2	27.5	46–70	
MPTP + HE3286	3	21	90.9	18.4	83–99	0.0001
MPTP + L-DOPA	2	15	83.8	10.6	78–90	0.05
MPTP + L-DOPA + HE3286	2	13	84.0	12.5	76–92	

^a^Dunn's post hoc test.
